# Dose Selection for Phase III Clinical Evaluation of Gepotidacin (GSK2140944) in the Treatment of Uncomplicated Urinary Tract Infections

**DOI:** 10.1128/aac.01492-21

**Published:** 2022-03-15

**Authors:** Nicole E. Scangarella-Oman, Mohammad Hossain, Jennifer L. Hoover, Caroline R. Perry, Courtney Tiffany, Aline Barth, Etienne F. Dumont

**Affiliations:** a Research and Development, GlaxoSmithKline plc, Upper Providence, Pennsylvania, USA

**Keywords:** antibacterial, dose selection, gepotidacin, uncomplicated urinary tract infection, acute uncomplicated cystitis, acute cystitis, pharmacokinetics, pharmacodynamics

## Abstract

Antibiotics are the current standard-of-care treatment for uncomplicated urinary tract infections (uUTIs). However, increasing rates of bacterial antibiotic resistance necessitate novel therapeutic options. Gepotidacin is a first-in-class triazaacenaphthylene antibiotic that selectively inhibits bacterial DNA replication by interaction with the bacterial subunits of DNA gyrase (GyrA) and topoisomerase IV (ParC). Gepotidacin is currently in clinical development for the treatment of uUTIs and other infections. In this article, we review data for gepotidacin from nonclinical studies, including *in vitro* activity, *in vivo* animal efficacy, and pharmacokinetic (PK) and pharmacokinetic/pharmacodynamic (PK/PD) models that informed dose selection for phase III clinical evaluation of gepotidacin. Based on this translational package of data, a gepotidacin 1,500-mg oral dose twice daily for 5 days was selected for two ongoing, randomized, multicenter, parallel-group, double-blind, double-dummy, active-comparator phase III clinical studies evaluating the safety and efficacy of gepotidacin in adolescent and adult female participants with uUTIs (ClinicalTrials.gov identifiers NCT04020341 and NCT04187144).

## INTRODUCTION

Urinary tract infections (UTIs) and their recurrence are associated with substantial health care burdens and morbidity globally, including impacts on the daily activities and quality of life of sufferers (1, 2). UTIs are defined as either uncomplicated (uUTIs) or complicated (cUTIs). uUTIs most frequently occur in females who are otherwise healthy, are not pregnant, are not immunocompromised, and have no abnormalities of the urogenital tract or signs of tissue invasion or systemic infection (3, 4). cUTIs are typically associated with men and with factors that compromise the urinary tract or host defense (3, 4).

The standard-of-care antibiotic treatment for uUTIs is one of several recommended agents: nitrofurantoin, trimethoprim, fosfomycin, or pivmecillinam (depending on regional availability), as first-choice agents, with fluoroquinolones or β-lactams as second choices (5). Selection of treatment is individualized and based on factors such as prior therapy, patient allergy and adherence history, local practice patterns, and local resistance data (5).

Antibiotic resistance among uropathogens causing uUTIs is increasing (3). Of great concern, UTIs caused by multidrug-resistant Gram-negative bacteria are increasing and are associated with high morbidity and mortality rates (6, 7). Escherichia coli is the most common causative uropathogen of uUTIs (8), with the proportion of uropathogenic E. coli isolates resistant to commonly prescribed antibiotics increasing markedly (9). Specifically, extended-spectrum β-lactamase (ESBL) production and fluoroquinolone resistance in uropathogenic E. coli from community-acquired UTIs are increasing (10, 11). Increasing rates of local antibiotic resistance, coupled with antibiotic allergy, intolerances, and contraindications, confer a threat to the use of oral antibiotics as an effective treatment for uUTIs in some patients, leaving fewer oral options and highlighting an unmet need for novel antibiotics to treat these infections (5).

One key consideration for antimicrobial drug development is the identification of optimal antimicrobial dosing regimens (dose and duration) to maximize the likelihood of antimicrobial success and resolution of infection (12). A regimen with a dose that is too low or a duration that is too short may result in incomplete bacterial eradication and encourage the development of resistance. Conversely, higher doses or longer durations may be less well tolerated, increase the risk of adverse events, impact the host’s internal flora, and increase the likelihood of infections caused by multidrug-resistant bacteria (12).

Gepotidacin (GEP) is a novel, first-in-class triazaacenaphthylene antibiotic that inhibits bacterial DNA replication by inhibiting DNA gyrase and topoisomerase IV by unique and balanced interactions on the GyrA subunit of bacterial DNA gyrase and the ParC subunit of bacterial topoisomerase IV in E. coli (13, 14). This distinct mechanism of action (13, 14) confers GEP activity against most strains of E. coli and Staphylococcus saprophyticus, even those that are resistant to current antibiotics such as fluoroquinolones (8, 15, 16). GEP previously demonstrated therapeutic efficacy in a phase IIa pharmacokinetic (PK)/pharmacodynamic (PD) study of 22 women with uUTI (8). GEP is currently being evaluated in phase III clinical trials for the treatment of uUTIs (ClinicalTrials.gov identifiers NCT04020341 and NCT04187144) (17, 18). The dose selection for GEP in these trials was guided by evaluations of its *in vitro* activity, *in vivo* efficacy, nonclinical PK/PD characterization in *in vitro* models, and clinical PK data from phase I and phase IIa studies.

In this review, we summarize how the results of these studies and analyses were used to select dosage regimens for the two ongoing GEP phase III studies in participants with uUTI.

## NONCLINICAL ANALYSES

### *In vitro* activity.

**FIG 1:**
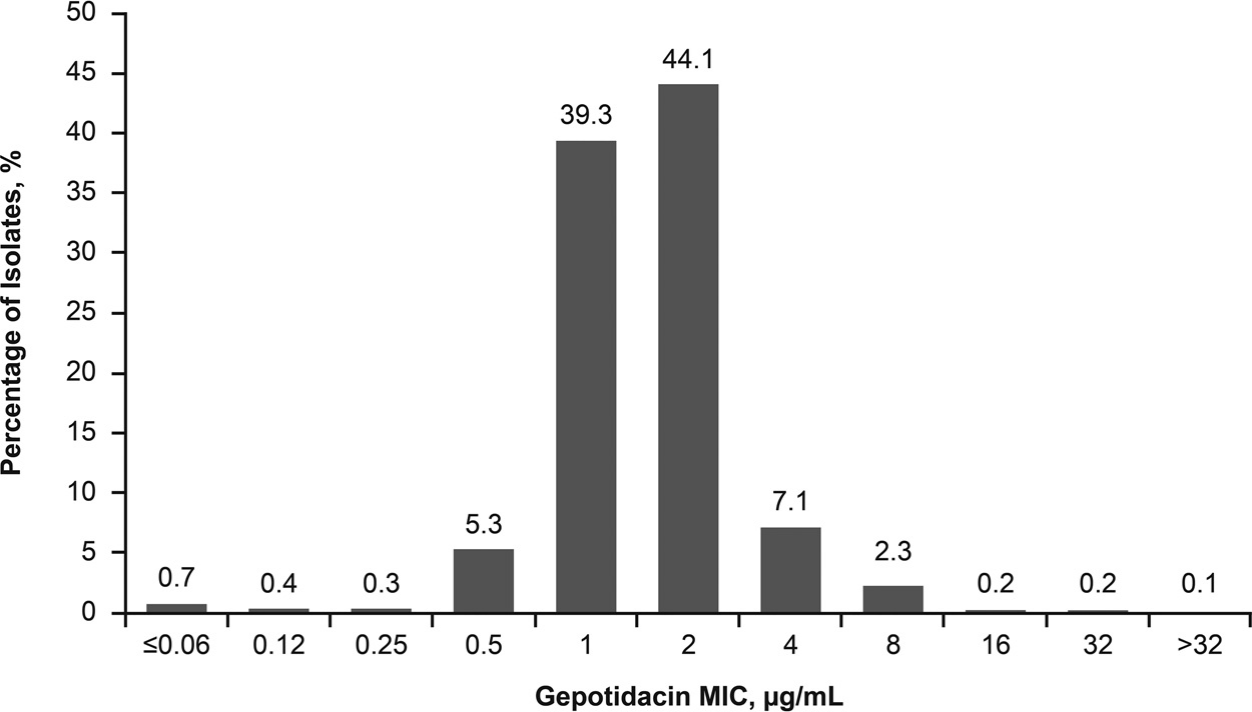
Frequency distribution of GEP MICs (micrograms per milliliter) against 1,010 E. coli isolates, including those resistant to levofloxacin (25.9% resistant isolates), fosfomycin (0.3%), nitrofurantoin (0.6%), or trimethoprim-sulfamethoxazole (37.7%) or positive for the ESBL phenotype (17.7%) (15). The ESBL phenotype was based on a ceftriaxone MIC of ≥2 μg/mL, according to CLSI guidelines (15, 33). (Based on data from reference 15.)

The *in vitro* activity of GEP was evaluated against a global collection of 1,010 E. coli isolates (15), measured by calculating GEP MICs using broth microdilution according to Clinical and Laboratory Standards Institute (CLSI) guidelines (15, 19). The frequency distribution of GEP MIC values for all 1,010 E. coli isolates is shown in Fig. 1. Overall, 97.2% (*n *= 982/1,010) of the tested isolates had GEP MIC values of ≤4 μg/mL (15). The MIC_90_ for GEP against all tested E. coli isolates was 2 μg/mL (*n *= 1,010) and increased to 4 μg/mL for ESBL-producing isolates (*n *= 179) and also for isolates not susceptible to nitrofurantoin (*n *= 55), fosfomycin (*n *= 11), or levofloxacin (*n *= 278) (15).

### *In vivo* efficacy in animal models.

Results from studies measuring the *in vitro* activity of antimicrobial agents do not always directly translate to the clinic, as such test systems are not able to fully simulate *in vivo* environments (20). The *in vivo* efficacy of antimicrobial agents also depends on their capacity to achieve an adequate concentration-time profile at the site of infection, i.e., urine/bladder (uUTIs) and kidneys (cUTIs) (21). Although cystitis models are the predominant animal model for studying uUTIs (22), uUTI and cystitis models in rodents can be variable and highly strain dependent, with marked self-resolution in untreated animals (23, 24). As such, a rat pyelonephritis (more indicative of cUTI) model was selected for the initial evaluation of the *in vivo* efficacy of GEP against E. coli (25). Although pyelonephritis is a more severe infection than cystitis, the model was chosen based on its robustness and reproducibility (25), and interpreting the results from this model in the context of uUTI can be considered conservative.

**FIG 2:**
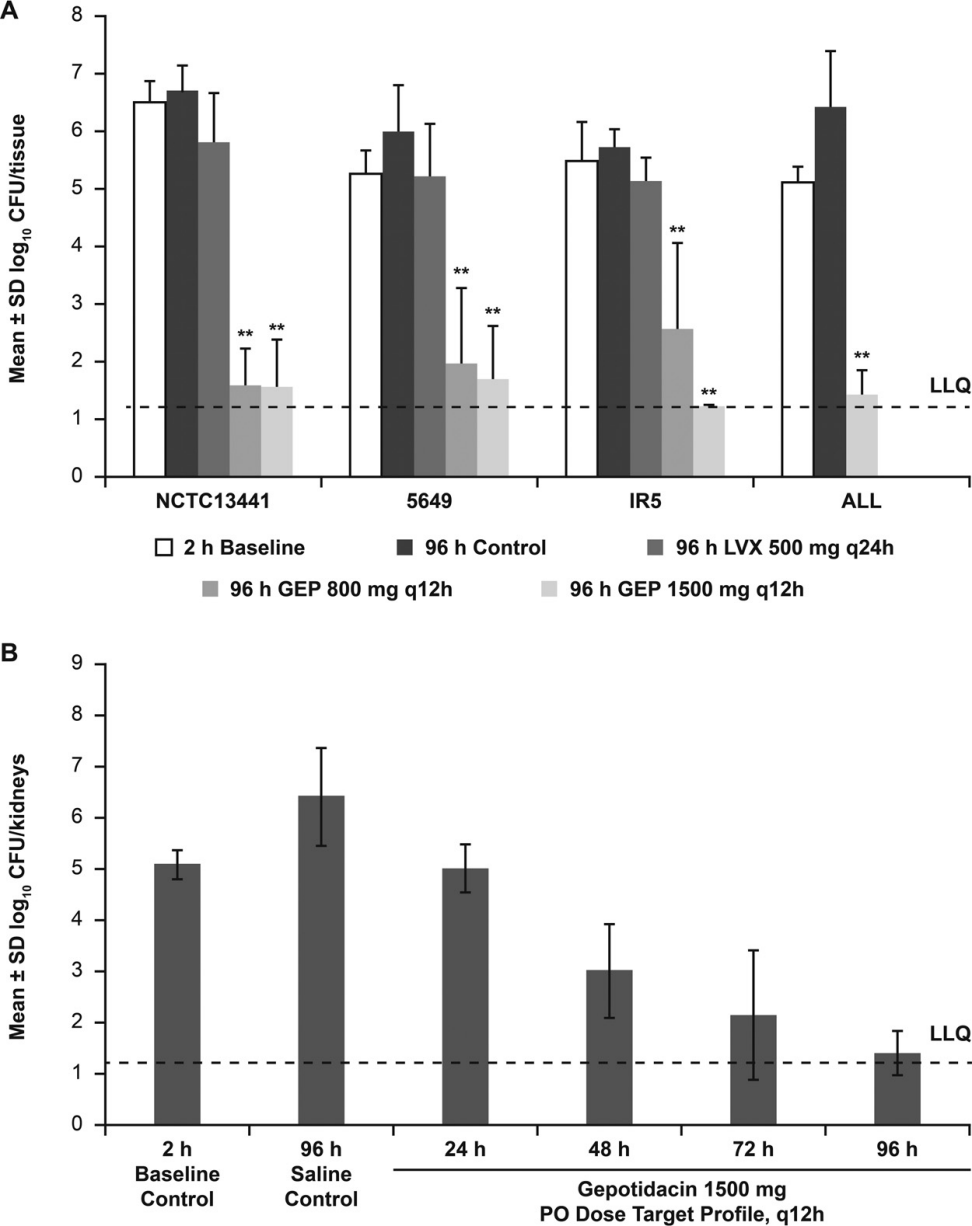
Reductions in bacterial burdens of four strains of multidrug-resistant E. coli after 4 days of treatment in rat kidneys (A) and time course of the efficacy of gepotidacin (GEP) at 1,500 mg q12h in rat kidneys (B) (25). Rats were infected by injection of E. coli directly into each kidney. At 2 h postinfection, the administration of gepotidacin was initiated using a continuous i.v. infusion into preimplanted jugular catheters. Infusion rates were controlled by preprogrammed infusion pumps, and flow rates varied over time to create systemic PK profiles in rats that mimicked systemic PK profiles measured in humans (both corrected for differences in protein binding); mean PK profiles from human oral doses of 800 mg and/or 1,500 mg q12h were tested. *, *P < *0.05; **, *P < *0.01 (representing a statistically significant reduction versus the 2-h baseline controls). Note that gepotidacin had previously demonstrated MICs of 2 to 4 μg/mL against the four strains of multidrug-resistant E. coli. LLQ, lower limit of quantification; LVX, levofloxacin; PO, oral; q12h, every 12 h; q24h, every 24 h. (Redrawn from reference 25.)

Using the rat pyelonephritis model, the efficacy of humanized PK exposures of GEP, recreated from mean PK profiles obtained with oral dosing regimens of 800 mg and 1,500 mg twice daily (BID) in healthy participants (in a randomized, single-blind phase I study) (26), was examined against four strains of multidrug-resistant E. coli (25). The four strains examined had GEP MICs of 2 μg/mL (strain 5649) or 4 μg/mL (strains IR5, ALL, and NCTC 13441). All strains were levofloxacin resistant (MIC of 16 or 32 μg/mL); one was of sequence type 131 (ST-131) (NCTC 13441), and three harbored New Delhi metallo-β-lactamase 1 (NDM-1) (5649, IR5 and ALL). Continuous intravenous (i.v.) infusion in rats was used to recreate mean human PK profiles for 800-mg and 1,500-mg oral GEP doses every 12 h (q12h). These doses were selected based on achieving a high probability of target attainment in patients (via Monte Carlo simulation) based on covering a GEP MIC of 4 μg/mL and an efficacious exposure range (free-drug area under the concentration-time curve [*f*AUC]) determined from previous *in vivo* efficacy studies (GlaxoSmithKline plc, data on file). Overall, treatment with both GEP exposures led to a reduced bacterial burden (log_10_ reduction in CFU per milliliter) compared with the baseline (nontreated) controls in the kidney (Fig. 2A) (25). The effectivenesses of the recreated GEP exposure profiles were not significantly different, although the 1,500-mg dose trended toward a (nonsignificant) greater reduction in the bacterial burden than the 800-mg dose (Fig. 2A). Similar results were observed in the bladder. Pooled mean daily blood AUC from 0 to 12 h (AUC_0–12_) values of GEP were 21 and 51 μg · h/mL (*f*AUC_0–12_, 14 and 34 μg · h/mL) in rat blood for the recreated human plasma PK 800-mg and 1,500-mg q12h oral doses, respectively. The mean GEP systemic exposure profiles in the rats were similar to the mean human exposure profiles for these doses (25).

Using the same rat pyelonephritis model, a separate time course experiment measured the reduction in bacterial burdens of multidrug-resistant E. coli following GEP exposure (25). Kidney and bladder CFU per milliliter were measured daily during q12h treatment targeting the recreated mean GEP 1,500-mg BID oral human exposure (25). A positive correlation between the duration of treatment and antibacterial efficacy was observed, with maximum efficacy and complete clearance of bacteria from both kidneys (Fig. 2B) demonstrated after 4 days (96 h) of treatment, with similar results observed in the bladder (25).

### Dynamic *in vitro* dose fractionation and dose-ranging studies.

*In vitro* and *in vivo* models are used to identify and predict optimal antibiotic dosing regimens that are most likely to demonstrate clinical efficacy and less likely to encourage resistance emergence (12, 27–29). *In vitro* dose fractionation and dose-ranging studies were conducted to determine the PK/PD characteristics of GEP against four well-characterized E. coli isolates (NCTC 13441 [ST-131 and cefotaxime-Munich-15 {CTX-M-15} resistance mechanisms], ALL [NDM-1, CTX-M-15, OXA-1, and OXA-2 β-lactamase resistance mechanisms], ATCC 25922 [wild type], and 3257 [CTX-M-15 resistance mechanisms]) using a one-compartment *in vitro* PK/PD model mimicking human free-drug plasma-concentration profiles. E. coli NCTC 13441 (GEP MIC of 2 μg/mL) was chosen based on its result reproducibility and representation of the ST-131 clonal group (30). The *in vitro* dose fractionation study demonstrated that the ratio of the area under the unbound drug concentration-time curve over 24 h at steady state to the MIC (*f*AUC_0–24_/MIC ratio) was the PK/PD index most predictive of GEP efficacy against E. coli (*r*^2^ = 0.925 for the *f*AUC_0–24_/MIC ratio, *r*^2^ = 0.874 for the ratio of the peak free-drug concentration to the MIC [*fC*_max_/MIC ratio], and *r*^2^ = 0.74 for the percentage of time that the free-drug concentration exceeded the MIC [*f*%T>MIC], versus the change from the baseline in log_10_ CFU per milliliter after 24 h of GEP therapy) (30). Of note, the *f*AUC_0–24_/MIC ratio has also been shown to be the PK/PD index most closely associated with GEP efficacy against other bacteria, specifically Staphylococcus aureus and Streptococcus pneumoniae (31). Dose-ranging studies of q12h human-simulated GEP (125 to 32,000 mg q12h over 1 day) were also performed. Across the four E. coli isolates (GEP MIC values of 1 to 4 μg/mL), the *f*AUC_0–24_/MIC values resulting in net bacterial stasis, a 1-log_10_ reduction, and a 2-log_10_ reduction in the bacterial burden from the baseline were 34.5, 41.3, and 49.7, respectively (30). Since a nonclinical pharmacodynamic target (PDT) associated with a 1-log_10_ reduction in the bacterial burden is typically selected for UTIs (27), 41.3 was selected as the subsequent PDT for GEP.

### *In vitro* hollow-fiber infection model.

**FIG 3:**
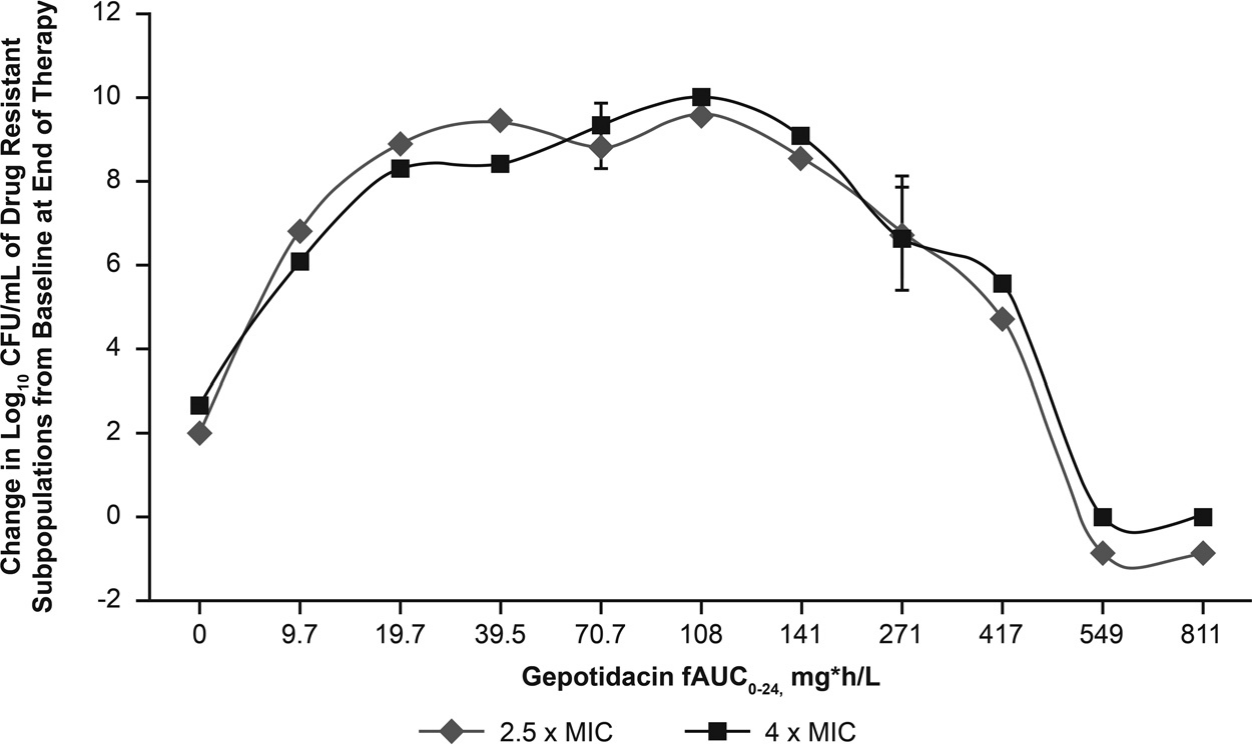
Relationship between gepotidacin exposure and change in log_10_ CFU per milliliter from the baseline of the gepotidacin-resistant subpopulation for E. coli NCTC 13441 on day 10. (Redrawn from reference 30.)

The predicted GEP exposure necessary to prevent resistance amplification was also assessed using a hollow-fiber infection model (30). A set of duplicate, 10-day, hollow-fiber infection model studies with the multidrug-resistant E. coli strain NCTC 13441 was used to determine the *f*AUC_0–24_/MIC exposure of GEP required to prevent the amplification of a resistant subpopulation. Using this approach, *f*AUC_0–24_ values of ≥549 μg · h/mL prevented resistance amplification to GEP for 10 days, equating to an *f*AUC_0–24_/MIC ratio of ≥275 when applying the GEP broth microdilution MIC of 2 μg/mL for E. coli NCTC 13441 (Fig. 3) (30). The preclinical data provided information on GEP exposures that may be needed to provide efficacy and suppress resistance amplification. While these data were a part of the data package used to further evaluate the potential of the 1,500-mg BID oral GEP dose for a 5-day dosing regimen in clinical studies of participants with uUTIs, the clinical significance and translation of these findings in the treatment of uUTI still need to be further understood.

## CLINICAL PHARMACOKINETIC/PHARMACODYNAMIC ANALYSES

### Human pharmacokinetics from phase I and phase IIa clinical studies.

The pharmacological activity of a drug depends on its adequate levels at the effect site. Plasma drug concentrations are often used as surrogate markers because access to effect sites for sampling can be limited (32). However, recent guidelines emphasize that PK from human studies should include data on drug exposure at the effect site (27). Furthermore, the CLSI and the European Committee on Antimicrobial Susceptibility Testing have approved breakpoints for several antimicrobial agents for the treatment of uUTIs to include isolates from urine only (33, 34). Urine sampling is relatively straightforward, and assessment of PK in urine provides insight into the likelihood of successful treatment of UTIs (32). To provide optimal GEP exposure data and a robust urine PK curve, extensive urine sampling was conducted to obtain PK data from healthy participants in phase I clinical studies (35–37) as well as from participants with uUTI in a phase IIa clinical study (8). Data from these studies were evaluated alongside the PK/PD targets identified from the previous *in vitro* studies to further support GEP dosing regimen selection for phase III clinical trials. A summary of the urine PK findings from the phase I and II studies is shown in Table 1. With respect to plasma PK, following the administration of single or repeat BID (400-, 800-, 1,500-, and 2,300-mg) or three-times-daily (TID) (1,500- and 2,000-mg) doses for 14 days (ClinicalTrials.gov identifier NCT01706315), GEP was rapidly absorbed, with a median time to maximum concentration of drug (*T*_max_) ranging from 1.25 to 2.5 h on day 1, and had an elimination half-life ranging from 11.2 to 14.7 h across all dose levels. Following 14 days of repeat BID or TID doses, the mean effective half-life values ranged from 6.12 to 8.96 h across all dose levels. The AUC_0–∞_ and *C*_max_ values increased in a greater-than-dose-proportional manner following single or repeat doses ranging from 400 to 2,300 mg (the slopes for *C*_max_ and AUC_0–∞_ were 1.38 and 1.30, respectively, for the day 1 single dose). Minimal to moderate accumulation was observed after repeat BID or TID dosing, and accumulation tended to be higher with TID dosing (accumulation ratios ranging between 1.27 and 1.52 for BID dosing and between 1.69 and 1.72 for TID dosing).

**TABLE 1:** Summary of GEP urine PK parameters in healthy participants and across clinical studies^*a*^

Parameter	Value for study									
	Phase I bioavailability study with healthy volunteers (*n* = 26) (ClinicalTrials.gov ID NCT02853435)^*c*^	Phase I study (ClinicalTrials.gov ID NCT03562117) for hepatic function^*d*^			Phase I study (ClinicalTrials.gov ID NCT02729038) for renal impairment^*e*^			Phase IIa uUTI study with participants with cystitis (*n* = 22) (ClinicalTrials.gov ID NCT03568942)^*f*^	Adult and adolescent study (administered with food) (ClinicalTrials.gov ID NCT04079790)^*g*^	
		Normal (*n* = 9)	Moderate (*n* = 8)	Severe (*n* = 8)	None (*n* = 8)	Moderate (*n* = 8)	Severe (*n* = 8)		Adults (*n* = 14)	Adolescents (*n* = 13)
Formulation	Tablet	Tablet	Tablet	Tablet	i.v.	i.v.	i.v.	Tablet	MS tablet	MS tablet
Dose	1,500 mg SD p.o.	1,500 mg SD p.o.			750 mg SD i.v.			1,500 mg p.o. BID	1,500 mg SD p.o.	1,500 mg p.o. BID
Urine PK sampling [no. of samples (sampling times)]	9 samples (hours 0 [predose],0–2, 2–4, 4–6, 6–8, 8–12, 12–24, 24–36, and 26–48)	9 samples (hours 0 [predose], 0–2, 2–4, 4–6, 6–8, 8–12, 12–24, 24–36, and 26–48)	6 samples (hours 0–6, 6–12, 12–24, 24–36, and 36–48)	6 samples (hours 0–6, 6–12, 12–24, 24–36, and 36–48)	6 samples (hours 0 [predose], 0–6, 6–12, 12–24, 24–36, and 36–48)			7 samples, with the 1st dose of GEP on day 1 and for the time-matched dose on day 4 (hours 0 [predose], 0–2, 2–4, 4–6, 6–8, 8–10, and 10–12)	9 samples (hours 0 [predose], 0–2, 2–4, 4–6, 6–8, 8–12, 12–24, 24–36, and 36–48)	9 samples (hours 0 [predose], 0–2, 2–4, 4–6, 6–8, 8–12, 12–24, 24–36, and 36–48)
Total unchanged GEP excreted in urine (mg)	∼287	113 (61.9)	168 (70.9)	299 (52.2)	280 (15.2)	166 (39.4)	59 (68.3)	460 (55.8)^*b*^	322 (19.7)	352 (25.5)
AUC_0–12_ (μg · h/mL) in urine	807	832 (118)	2,164 (131)	3,285 (110)	2,426 (20.6)	1,608 (42.7)	512 (50.0)	–	–	–
AUC_0–24_ (μg · h/mL) in urine	–	938 (105)	2,274 (105)	4,247 (99.1)	2,743 (20.1)	1,808 (39.0)	619 (49.6)	11,945 (87.2)^*b*^	2,750 (69.4)	3,660 (87.2)
AUC_0–48_ (μg · h/mL) in urine	1,382	991 (114)	3,162 (61.9)	3,902 (81.3)	2,941 (21.2)	1,827 (38.6)	682 (47.9)	–	2,980 (67.0)	4,070 (84.7)
CLr (L/h)	–	7.59 (46.6)	9.08 (32.8)	11.8 (38.7)	19.2 (18.8)	7.6 (44.3)	2.1 (84.3)	15.7 (45.2)^*b*^	16.4 (19.6)	15.1 (25.9)
*fe*%	–	7.53 (61.9)	11.2 (70.9)	19.9 (52.2)	37.4 (15.2)	22.1 (39.4)	7.9 (68.3)	30.7 (55.8)^*b*^	21.5 (19.7)	23.4 (25.5)

a Values are presented as geometric means (%CVb [percent between-participant geometric coefficient of variation]) unless otherwise stated. AUC, area under the concentration-time curve (subscripts indicate time ranges); BID, twice daily; CLr, renal clearance; *fe*%, percentage of the given dose excreted in urine; MS, mesylate salt; p.o., oral; SD, single dose; i.v., intravenous. –, not applicable.

b Geometric mean (%CVb) values on day 4.

c See reference 35.

d See reference 38.

e See reference 37.

f See reference 8.

g See reference 36.

The study under ClinicalTrials.gov identifier NCT04079790 (36) was conducted to assess systemic exposure in adults and adolescents, with one aim of supporting the inclusion of adolescents ≥12 years of age in phase III clinical development (Table 1). Although PK parameters were generally similar among adolescents and adults receiving the single 1,500-mg dose, adolescents recorded higher AUC_0–24_ and AUC_0–48_ values in urine than those of adults.

GEP exposure in the urine was also examined in a phase I study (ClinicalTrials.gov identifier NCT03562117) in participants with hepatic impairment (Table 1). Values for GEP urinary exposure and the total amount excreted into the urine increased with increasing severity of hepatic impairment (Table 1) (38). Urine GEP concentrations remained high for 12 h postdose in participants with normal and impaired hepatic function.

GEP exposure in urine was also examined in a phase I study (ClinicalTrials.gov identifier NCT02729038) in participants with renal impairment (Table 1) (37). Overall, the urine GEP exposure, renal clearance, and concentration excreted were lower with decreased renal function, and the study concluded that patients with severe renal impairment may require dose adjustment or adjustments to the dosing frequency.

Furthermore, an absorption, distribution, metabolism, and excretion study for GEP describing GEP PK in healthy subjects indicated that when administered as a single i.v. dose, approximately 60% of GEP was recovered in urine (39). In addition, renal clearance of GEP was approximately 40% of the total systemic drug clearance. These results indicate that renal impairment may have the potential to adversely affect the elimination of GEP.

**FIG 4:**
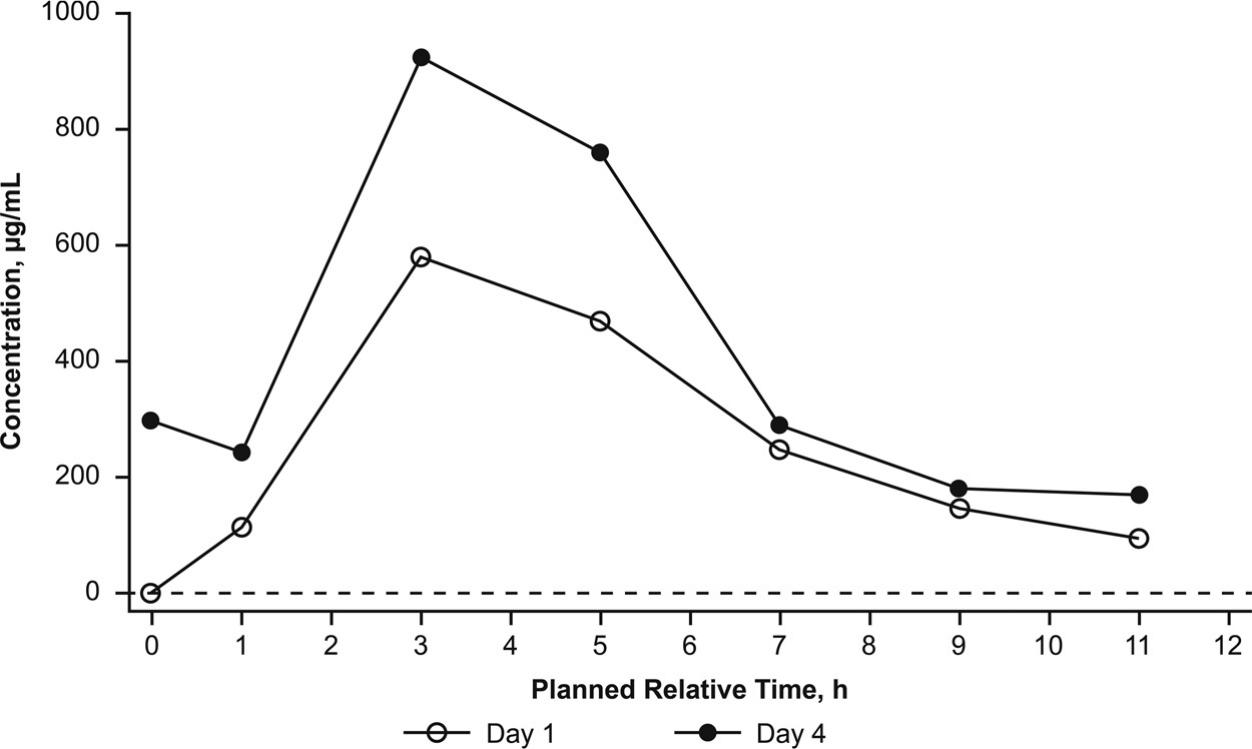
Median gepotidacin urine concentration-time plot following oral administration of gepotidacin at 1,500 mg BID from the phase IIa uUTI study (ClinicalTrials.gov identifier NCT03568942) in 22 participants with uUTIs (8). The lower limit of quantification, represented by the dashed line, was 1.00 μg/mL. Data are plotted by the planned relative midpoint time for each interval. Notes that based on trough predose plasma concentrations of gepotidacin and statistical analysis, plasma steady state was achieved by day 3. One participant received nine doses of the study treatment. The second dose of the study treatment was not administered due to a study treatment administration protocol deviation by the site. (Redrawn from reference 8.)

In the phase IIa study (ClinicalTrials.gov identifier NCT03568942) to evaluate plasma and urine PK, safety, and exploratory efficacy endpoints, participants with uUTIs received oral GEP at 1,500 mg BID for 5 days in the clinic (on-therapy period; days 1 to 5) and returned to the clinic for test-of-cure (days 10 to 13) and follow-up (day 28 ± 3) visits (8). In the total population, the plasma steady-state concentrations of GEP were achieved on day 3, and the minimum steady-state plasma AUC_0–12_ on day 4 was 15.2 μg · h/mL (8). Median urine GEP concentrations were generally higher on day 4 than on day 1 (Fig. 4). The geometric mean minimum steady-state urine AUC_0–τ_ and AUC_0–24_ values on day 4 were 2,256 μg · h/mL and 4,512 μg · h/mL, respectively (8). Steady-state total drug urine trough levels remained above the GEP MIC of 4 μg/mL throughout the 12-h dosing interval (8) and were higher than the GEP MIC_90_ values for common uUTI pathogens such as quinolone-resistant E. coli (MIC_90_ of 4 μg/mL) (15). GEP at 1,500 mg BID provided >600-fold-higher total drug concentrations (day 3 urine *C*_τ_ [plasma predose concentrations]/day 3 free-drug plasma *C*_τ_) in urine than the free-drug concentration in the plasma at steady state, demonstrating increased exposures at the target infection site (urine/bladder) (8).

## CLINICAL EFFICACY FROM A PHASE IIa CLINICAL STUDY

Of 22 participants in the phase IIa study (ClinicalTrials.gov identifier NCT03568942), 8 had baseline isolates (5 E. coli and 1 each of Citrobacter koseri, Klebsiella pneumoniae, and S. saprophyticus) that were qualifying uropathogens (growth, ≥10^5^ CFU/mL) and thus were included in the microbiological intent-to-treat population (8). Against these eight qualifying uropathogens, GEP MIC values ranged from 0.06 to 4 μg/mL.

The exploratory efficacy of GEP was determined in the total population (*N *= 22) and in the microbiological intent-to-treat population (*n *= 8) (8). Clinical success was defined as the resolution of signs and symptoms present at baseline (and no new signs and symptoms) by the test of cure (days 10 to 13) and no required use of any other antimicrobial therapy for the current uUTI. For the total population, clinical success at the test-of-cure and follow-up visits was achieved for 19/22 participants (86% [Clopper-Pearson 95% confidence interval {CI}, 65% to 97%]) and 18/22 participants (82% [Clopper-Pearson 95% CI, 3% to 35%]), respectively (8). Of the participants with qualifying uropathogens, clinical success was achieved for 7/8 (88% [Clopper-Pearson 95% CI, 47% to >99%]) at both the test-of-cure and follow-up visits. Microbiological success, defined as culture-confirmed eradication (no growth [<10^3^ CFU/mL]) of the baseline uropathogen, was also achieved in 7/8 (88%) participants at the test of cure (the failure was due to an indeterminant laboratory result, but the participant was a clinical success), with 6/8 (75%) with success at follow-up (8). There was no persistence (0/8 participants) of the baseline uropathogen at the test-of-cure visit or a reduction in susceptibility to GEP (i.e., a ≥4-fold increase in the MIC for uropathogens of the same species) at any time during the study period.

### Human pharmacokinetics/pharmacodynamics from phase I and phase IIa clinical studies.

The human urine exposures reported in the phase I and IIa studies were higher than the GEP concentrations required for efficacy or prevention of resistance in the nonclinical *in vitro* models (30). The AUC/MIC ratio achieved in urine was calculated by applying the human PK data from these clinical studies (e.g., urine AUC) and dividing by an MIC of 4 μg/mL, which represents the MIC_90_ for fluoroquinolone-resistant and ESBL-producing E. coli in a large MIC study (Table 2) (40). For the phase I study’s (ClinicalTrials.gov identifier NCT02853435) 26 healthy volunteers, receiving a single dose of GEP of 1,500 mg (40), the calculated total urine AUC_0–24_/MIC ratio (urine exposures are not adjusted for protein binding) of 403 exceeded the target for delivering efficacy at the site of infection (*f*AUC_0–24_/MIC magnitude required for a 1-log reduction in the bacterial burden *in vitro*) (30) by approximately 10-fold and the potential target for resistance suppression (as determined from an *in vitro* PK/PD hollow-fiber model) by approximately 1.5-fold (40). For the phase IIa study’s (ClinicalTrials.gov identifier NCT03568942) participants, the calculated urine AUC_0–24_/MIC ratio of 1,128 exceeded the same nonclinical PK/PD targets for efficacy and resistance suppression (30) by approximately 27-fold and 4-fold, respectively (40). Thus, the minimum urine AUC_0–24_ for the 1,500-mg oral BID dose determined in healthy volunteers and separately in participants with uUTI exceeded the *in vitro* nonclinical efficacy and resistance suppression PK/PD targets derived from the nonclinical models.

**TABLE 2:** Summary of GEP human urine exposures and nonclinical *in vitro* PK/PD targets^*e*^

Item (reference[s])	AUC_24_ (μg · h/mL)^*a*^	*f*AUC_0–24_/MIC ratio
Efficacy target (*f*AUC_0–24_/MIC magnitude required for a 1-log reduction in bacterial burden) from E. coli *in vitro* PK/PD model (30)	–	41.3
Resistance suppression target from a 10-day E. coli hollow-fiber infection model (30)	–	275
Minimum urine exposure from healthy volunteers following single oral dose of GEP at 1,500 mg BID (35, 40)	1,614^*b*^	403^*a*^^,^^*c*^
Steady-state (day 4) minimum urine concn from participants with uUTI^*d*^ following oral GEP at 1,500 mg BID for 5 days (total of 10 doses) (8)	4,512	1,128^*a*^^,^^*c*^

a Protein binding adjustment was not required for urine. –, not applicable.

b Calculated from a minimum urine AUC_12_ of 807 μg · h/mL.

c A GEP MIC of 4 μg/mL was applied (gepotidacin MIC_90_ against fluoroquinolone-resistant E. coli) (15).

d Twenty-two participants were evaluated (8).

e AUC, area under the concentration-time curve; BID, twice daily; GEP, gepotidacin; PD, pharmacodynamics; PK, pharmacokinetics; uUTI, uncomplicated urinary tract infection.

Of the eight qualifying uropathogens from the participants in the phase IIa study (ClinicalTrials.gov identifier NCT03568942), six had both available PK and a qualifying *Enterobacterales* uropathogen. For these participants, plasma *f*AUC_0–24_/MIC ratios ranged from 6.99 to 90.5, and urine AUC_0–24_/MIC ratios ranged from 1,292 to 121,698 (8). For one of these participants, GEP demonstrated microbiological success in treating a participant with a K. pneumoniae qualifying uropathogen with an MIC of 4 μg/mL, at a plasma *f*AUC_0–24_/MIC ratio of 6.99 and a urine AUC_0–24_/MIC ratio of 1,292 (8). In the four participants with qualifying E. coli uropathogens and who were microbiological successes, urine AUC_0–24_/MIC ratios ranged from 1,292 to 121,698 (8), equating to ∼31- to 2,947-fold above the nonclinical efficacy PK/PD target and ∼4.7- to 443-fold above the nonclinical resistance suppression PK/PD target.

## SUMMARIZING DOSE REGIMEN SELECTION FOR PHASE III STUDIES IN UNCOMPLICATED URINARY TRACT INFECTIONS

The selection of the optimal GEP dosing regimen for phase III uUTI clinical studies was founded on the comprehensive body of data derived from the nonclinical studies and early-phase clinical studies discussed above. Evaluation of GEP’s *in vitro* activity against over 1,010 E. coli isolates identified an MIC_90_ for GEP of 2 μg/mL (15), while *in vivo* animal studies showed maximal GEP efficacy in the kidneys against multidrug-resistant E. coli with MICs up to and including 4 μg/mL after 4 days of treatment with humanized exposures mimicking an oral GEP 1,500-mg BID dose (25). *In vitro* dose fractionation and dose-ranging studies revealed that the *f*AUC_0–24_/MIC ratio was the PK/PD index that best predicted GEP efficacy, and the magnitude of this index for a 1-log reduction in the bacterial burden was 41.3 (30). An *f*AUC_0–24_/MIC ratio of 275 prevented resistance amplification in an *in vitro* hollow-fiber model (30). Finally, data from phase I and phase IIa clinical studies with GEP oral dosing of either a single dose or 5 days BID of 1,500 mg showed high GEP exposures in urine in both healthy volunteers and participants with uUTIs (8, 35, 36, 38, 41), with the minimum observed GEP urine levels resulting in urine AUC_0–24_/MIC values exceeding the model-derived nonclinical efficacy and resistance suppression PK/PD targets. The clinical significance and translation of these findings in the treatment of uUTI still need to be further understood. The clinical and microbiological efficacy of GEP was also demonstrated in the phase IIa study (8). GEP administered as a 1,500-mg BID oral dose for 5 days was selected as the optimum dosing regimen for two currently ongoing phase III studies (ClinicalTrials.gov identifiers NCT04020341 [17] and NCT04187144 [18]) in participants with uUTI.

## LIMITATIONS OF PRECLINICAL AND CLINICAL STUDIES

Our studies had several limitations. Only one isolate was evaluated in the *in vitro* hollow-fiber infection model; therefore, it was not possible to evaluate interisolate variability for the exposure associated with resistance prevention (30). The clinical significance and translation of this model to predict resistance suppression in the treatment of uUTI still need to be established and further understood. A rat pyelonephritis was used rather than a cystitis model; however, as pyelonephritis is a more severe infection than cystitis, the use of this model in the context of uUTI can be considered conservative. Additionally, the bacterial load was measured in the kidney and bladder in this model rather than urine, which is used to determine microbiological outcomes in the clinical setting.

The phase I and phase IIa studies were open-label, noncomparative studies. The phase IIa study was a single-center study in the United States, and because sampling was limited to one site, there were few drug-resistant isolates for evaluation. Furthermore, the sample size for microbiological assessment was small in this study, as few pathogens met the regulatory qualifying uropathogen growth criteria. Our approach for dose selection diverged from the use of drug concentrations in the plasma; however, the use of drug concentrations in urine is in line with recent guidance on the importance of considering exposures at the target site of infection and the number of approved antibacterials with breakpoints specific for urinary tract infections (32–34). Consideration of GEP for future studies evaluating the treatment of infections at other body sites would require different PK considerations and is beyond the scope of this uUTI-focused summary.

## CONCLUSION

In the GEP phase III clinical studies, urine levels of GEP observed following a 1,500-mg BID oral dose are anticipated to exceed the *f*AUC/MIC nonclinical efficacy and resistance suppression PK/PD targets for GEP against E. coli determined from *in vitro* PK/PD models. Given that the bladder is the primary site of infection in uUTIs, a translational package combining GEP urine PK and PK/PD data, *in vitro* activity data, *in vivo* efficacy in a robust humanized PK pyelonephritis model, and confirmatory clinical and microbiological findings from a phase IIa uUTI study was used for the selection of the GEP dose and duration. A GEP 1,500-mg BID oral dose for 5 days was thus selected for the treatment of participants with uUTIs in two currently ongoing phase III clinical studies (ClinicalTrials.gov identifiers NCT04020341 and NCT04187144). Additional data derived from these studies will allow the further study of the GEP dose regimen and a greater understanding of GEP’s PK variability in larger participant cohorts.
